# Microencapsulated diets to improve bivalve shellfish aquaculture

**DOI:** 10.1098/rsos.171142

**Published:** 2017-11-08

**Authors:** David Willer, David C. Aldridge

**Affiliations:** Department of Zoology, University of Cambridge, The David Attenborough Building, Pembroke Street, Cambridge CB2 3QY, UK

**Keywords:** microencapsulated diet, bivalve shellfish, aquaculture, BioBullets, nutrition, mussels

## Abstract

Aquaculture is the fastest growing food sector and feeds over 3 billion people. Bivalve shellfish aquaculture makes up 25% of global aquaculture production and is worth annually US$19 billion, but continued growth is currently limited by suboptimal diets and limited tools for disease control. New advances in microencapsulation technology could provide an effective way to overcome these biological limitations. This study demonstrated that a new formulation of microencapsulated diet known as BioBullets could be ingested by a commercially farmed bivalve; the blue mussel *Mytilus edulis*. Microparticles could be captured by mussels with similar efficiency to natural foods. Microparticles too large for ingestion were rejected in pseudofaeces. Microparticles were successfully ingested and broken down by the gut. Further work is needed to assess the impact of BioBullets diets on bivalve growth. There is now an exciting opportunity to tailor the composition of microencapsulated diets for specific applications to improve production output and efficiency in the commercial bivalve shellfish industry.

## 1. Introduction

Aquaculture is the fastest growing food sector and continues to expand alongside terrestrial crop and livestock production [1]. Globally over 3 billion people depend on aquaculture for at least 20% of their dietary protein [1]. Expanding aquaculture has been identified as a critical component in securing food for 9 billion people by 2050 [2]. Bivalve shellfish aquaculture makes up nearly 25% of global production, and over the last decade has grown at 10% per year to a value of US$19 billion in 2014 [3]. However, the continued growth of bivalve aquaculture is limited by suboptimal feeding protocols and disease control methods. Currently, lipid-rich cultures of microalgae are grown to feed bivalves while they are in the hatcheries. However, growing these cultures is expensive, equating to over 30–50% of total production costs [4], and cultures are highly subject to contamination and have variable nutritional value [5]. Bivalve aquaculture suffers also high losses due to frequent outbreaks of infectious disease [6]. New advances in microencapsulation technology through chemical engineering can provide a way to overcome current biological limitations in bivalve shellfish production. A novel form of microparticles have recently been developed by BioBullets (BioBullets Ltd, Cambridge, UK) for the targeted delivery of chemical control agents to invasive bivalve species [7]. The BioBullets delivery system is highly effective and cheap to manufacture, opening a new direction for this emerging technology in feeding desirable products to bivalves.

Microencapsulation technology offers many critical advantages over alternative strategies that could be used to tackle production limitations. First, a single microencapsulated feed particle can contain an optimal formulation of key nutrients for bivalve growth, specifically high levels of protein and polyunsaturated fatty acids including docosahexaenoic acid (DHA) and eicosapentaenoic acid (EPA), alongside disease control agents. The microencapsulated diet could be tailored to specific species, or specific geographies where certain nutrients are lacking. Even the highest quality live microalgae will rarely have an optimal nutrient composition [8], and so currently multiple cultures of different microalgal species are grown, which increases production costs and commercial risk in the case of contamination. Second, maximal particle capture can be ensured by customizing microparticle size and buoyancy. Size can be tailored to bivalve preferences for maximum retention efficiency, and buoyancy can be optimized to ensure particles remain within reach of feeding bivalves [5]. This is a key advantage over nutrient delivery systems such as powders, which tend to float on the water surface, and can clump into particles too large to be retained by bivalves. Third, preingestive nutrient loss can be minimized by using an encapsulant that retains particle nutrients until they are released by the digestive processes of the bivalve. Lipid coatings allow delivery of low molecular weight, water soluble compounds with minimal leaching to the surrounding water [9]. Fourth, storage of high quality feeds for long time periods is made possible by the stable nature of microparticles in air [5]. This is a major advantage over simply growing higher quality algal cultures; stored microparticles are less likely to become contaminated over time by bacteria than live algal cultures, and the costly process of synchronizing microalgal feedstock with bivalve production is avoided [5]. Therefore, new microencapsulation technology can offer an efficient way to deliver replacement or supplementary diets that could improve bivalve nutrition, growth, and production output, while reducing bivalve mortality, production costs, and financial risks.

The capture and ingestion of food particles by bivalves in aquaculture or the wild is highly influenced by particle characteristics. Food particles are collected through the inhalant siphon and sorted on the labial palps and gills using size, shape and other physical attributes. Unwanted particles, particularly sharp edged or inorganic particles such as SiO_2_, are preferentially rejected in pseudofaeces. Larger bivalves including oysters ingest organic particles up to 400 µm diameter. Blue mussels (*Mytilus edulis*) will select diatoms and dinoflagellates up to 200 µm [10]. Particle size selectivity increases when food is more plentiful; blue mussels will preferentially select particles of between 20 and 40 µm, and reject a greater proportion of particles larger than 40 µm as food availability increases [10,11]. Once ingested, food particles are swept along ciliated sorting areas in the stomach towards the digestive glands. Particles too large for digestion are rejected down deep grooves in the stomach to the intestine, although a small number to remain aid food grinding in the stomach [10,12]. Any microencapsulated diet fed to bivalves will be subjected to these same sorting processes, and therefore the development of effective microencapsulated diets requires a sound understanding of microparticle ingestion by bivalves.

**Figure 1.**
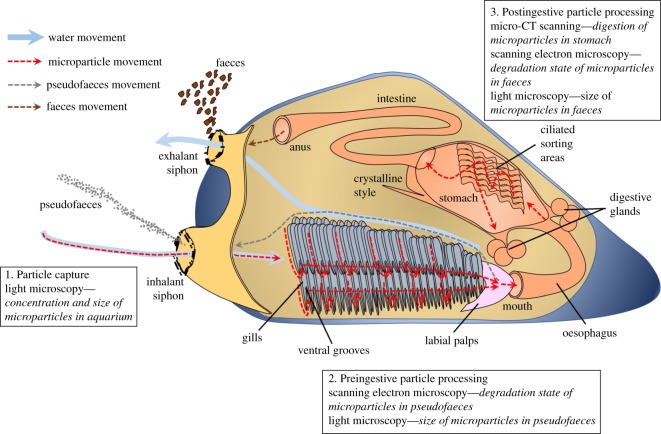
Schematic diagram of microparticle digestion in the blue mussel *Mytilus edulis*. The boxes outline the analytical techniques used to characterize each digestion stage investigated during the study; particle capture, and preingestive and postingestive particle processing.

This study aimed to demonstrate that a new form of BioBullets microparticles containing a microalgal food could be successfully captured and ingested by a commercially farmed bivalve; the blue mussel *Mytilus edulis*. Blue mussels have great commercial importance, and with a value of US$3.6 billion make up 20% of the global bivalve aquaculture market [13]. In 2015 2.3 million tonnes were produced, primarily in coastal Western Europe and Canada. Three components of microparticle digestion were investigated as outlined in figure 1. Firstly, particle capture, to assess whether and how rapidly microparticles could be cleared from water by mussels. Secondly, preingestive particle processing, to assess which sizes of microparticles were preferentially ingested or alternatively rejected in pseudofaeces. Thirdly, postingestive particle processing, to confirm successful ingestion and assess how well microparticles were broken down by the gut. By demonstrating successful ingestion of BioBullets microparticles, this study opens a gateway to further optimize the composition of microparticles for specific applications in bivalve aquaculture.

## 2. Methods

### 2.1. Microparticle production

Lipid-walled microparticles containing *Schizochytrium* microalgae were manufactured by BioBullets Ltd (Cambridge, UK). A premix slurry was prepared containing the encapsulant and powdered microalgae under conditions of controlled shear. The slurry was pumped into an ultrasonic atomizing nozzle at the top of a cooling chamber. The atomized particles formed perfect spheres as they cooled and fell to the chamber base. Further particle cooling was achieved with an air-conveying system before discharge via cyclone to a fluid bed processor. The encapsulated particles were then coated with a proprietary non-ionic surfactant to aid dispersion in water. Further cooling in the fluid bed removed all heat of crystallization from the microparticles before packaging.

### 2.2. Mussel husbandry

The blue mussel *Mytilus edulis* was selected as the model bivalve species to establish whether BioBullets microparticles could be successfully digested. Mussels were collected at low tide from the shore at Old Hunstanton, Norfolk, England (52°56′56 N, 0°29′27 E) on 1 February and 1 March 2017. Mussels were kept in constantly aerated aquaria at 10°C in seawater from Old Hunstanton with a salinity of 32 ppt.

### 2.3. Particle clearance

To investigate particle clearance, five aquaria aerated at a constant rate were set up with 200 ml seawater from the collection site. A single mussel was measured in length and wet mass, and added to each aquarium, then left for 5 days to allow acclimation and byssus thread attachment. The mussels were large adults with a length of 35–40 mm. To provide a starting concentration of 50 mg l^−1^, 10 mg dry weight microparticles (90 000 microparticles) were then added to each aquarium. Next, 1 ml water samples were collected every 5 min, using a pipette with a modified 5 mm aperture tip to allow particle entry. Each water sample was mixed and placed on a 1 ml Sedgewick Rafter counting cell, and the number of microparticles in the central 25 mm^2^ grid counted under a dissecting microscope, providing a microparticle concentration per 25 µl. The sampling process was repeated for three control aquaria, containing a starting concentration of 50 mg l^−1^ microparticles, but no mussels.

### 2.4. Particle size selectivity

To assess particle size selectivity, eight aerated aquaria were set up with 200 ml seawater. A single mussel was added to each aquarium, along with 50 mg microparticles to provide a starting concentration of 250 mg l^−1^. The mussels were large adults with a length of 35–40 mm. Mussels were left for 4 days to allow pseudofaeces and faeces production. Pseudofaeces thread samples were then collected from outside the inhalant siphon of each mussel using tweezers, and faecal pellet samples from near the exhalant siphon, in sufficient quantities to fill a 9 mm^2^ grid area for each sample. For each sample the diameter of every microparticle within the 9 mm^2^ area was measured to the nearest 5 µm under light microscopy using a slide graticule and eyepiece micrometer. The measuring process was repeated with 3 control samples of microparticles in seawater in the absence of mussels.

### 2.5. Particle imaging

Particles were imaged using scanning electron microscopy (SEM) to look for and assess the degradation state of microparticles in faeces and pseudofaeces. Micro computed tomography (micro-CT) scanning was used to examine microparticle digestion within the mussel gut cavity.

To obtain samples for SEM imaging, two aquaria were set up with 1 l seawater and 10 mussels each, left for 7 days to allow any existing food to be purged from the mussel digestive tract, then 500 mg microparticles were added to one aquarium. Mussel pseudofaeces and faecal pellets were collected from both aquaria after 3 days. The mussels were placed in fresh water at the point of pseudofaecal and faecal sampling to wash the salt from the pseudofaeces. The salt was removed from faecal pellets using a centrifuge at 1000 g for 30 s and three washes of deionized water. Pseudofaeces and faeces samples were freeze-dried overnight, mounted on SEM stubs with silver paint, and sputter-coated with 30 nm carbon. To allow for an assessment of the degradation of particles that had been processed by the mussels, a sample of unfed microparticles was also freeze dried, mounted and carbon-coated. SEM images were then taken using an FEI Verios 460 (Thermo Fisher Scientific, USA).

To prepare for the micro-CT scanning, a 1 l seawater aquarium with 10 mussels of length 40 mm was treated with 500 mg microparticles for 16 h. Four mussels which had been observed feeding regularly were then selected, and after removing one shell half these were fixed in 4% formaldehyde overnight. The fixative was removed with three 10 min washes in water and the mussels were then sequentially dehydrated in 25, 50, 75 and 100% methanol for 15 min each. The mussels were then immersed in a 1% phosphotungstic acid (PTA) in methanol stain for 10 days, with the staining solution changed after 5 days. This process was then repeated with two mussels that had not been fed microparticles as a negative control. Mussels were mounted and then scanned at the Cambridge Biotomography Centre using a Nikon XT 225 ST micro-CT scanner; the settings were 120 kV and 130 μA. The images were constructed from 1080 projections each with 1000 ms exposure. ImageJ [14] was used to process the images and search for microparticles within individual scan slices of the mussel gut.

### 2.6 Data analysis

The rate of particle clearance was normally distributed. A linear model and subsequent ANCOVA was used to compare the change in log_10_ microparticle concentration over time between mussels and control samples. Clearance rates of microparticles were calculated per gram wet mussel mass per minute, and the background decline in microparticle concentration was accounted for by subtracting the mean decline in microparticle concentration in control samples from each clearance rate. ANOVA was used to assess the change in clearance rate as microparticle concentration declined over time.

To investigate particle selectivity, pairwise comparisons among least-squares means were used to test for differences in particle size distribution between microparticles from faeces, pseudofaeces and unfed samples. Differences in mean microparticle diameter between all three sample types were assessed using Kruskal–Wallis with *post hoc* Dunn's tests.

Data were analysed using the statistical package R [15].

## 3. Results

### 3.1. Particle capture

**Figure 2.**
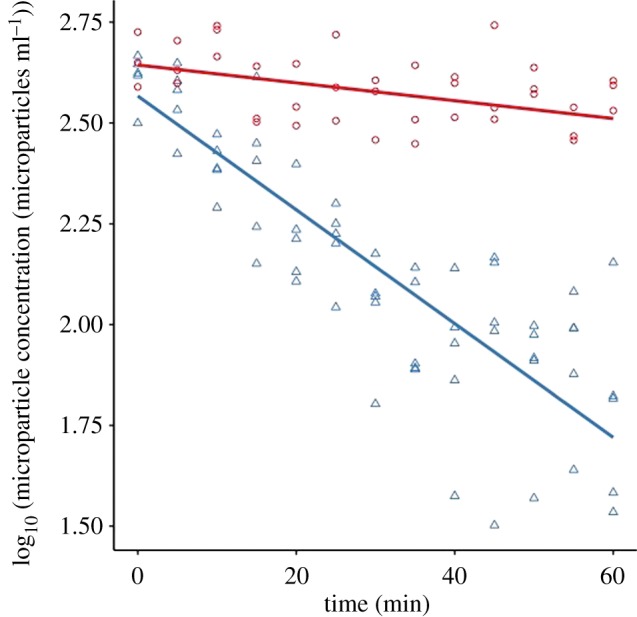
Comparison of microparticle concentration over time between mussel (blue triangles) and control (red circles) aquaria. Ten milligrams of microparticles were added to 200 ml aquaria containing one mussel or no mussel for the controls, and the microparticle concentration was recorded every 5 min for 60 min. The decline in microparticle concentration in mussel aquaria was significantly greater than the decline in control aquaria (ANCOVA, *p* < 0.001). Sample sizes: 5 mussels of length 35–40 mm, 3 controls. Regression lines are linear models of log_10_ microparticle concentration on mussels or controls. For mussels *y *= 2.57 − 0.014*x*, *r*^2^ = 0.78, *p* < 0.001; for controls *y *= 2.64 − 0.0022*x*, *r*^2^ = 0.38, *p* < 0.001.

Microparticles were cleared rapidly from seawater by mussels. The mean microparticle concentration in the aquaria declined by 83.6 ± 6.4% over a 60-min period, from 440.0 ± 28.3 (standard error) to 72.0 ± 15.0 microparticles ml^−1^ (figure 2). This decline was significantly greater than in control aquaria without mussels (figure 2), where microparticle concentration declined slowly from 466.7 ± 26.7 to 306.7 ± 13.3 microparticles ml^−1^ (ANCOVA, *F*_3,100_ = 222, *p* < 0.001). Accounting for this background settlement, the mean decline in microparticle concentration in aquaria with mussels was 52.7 ± 6.4%. The mean initial clearance rate over the first 5 min was 0.970 ± 0.326 microparticles ml^−1^ g^−1^ wet mussel mass min^−1^. Clearance rates fell significantly over time to a final value at 60 min of 0.051 ± 0.278 microparticles ml^−1^ g^−1^ wet mussel mass min^−1^ as microparticle concentration in the water declined (ANOVA, *F*_1,58_ = 13.9, *p* < 0.001, comparison between 5 and 60 min with correction for settling).

### 3.2. Preingestive particle processing

**Figure 3.**
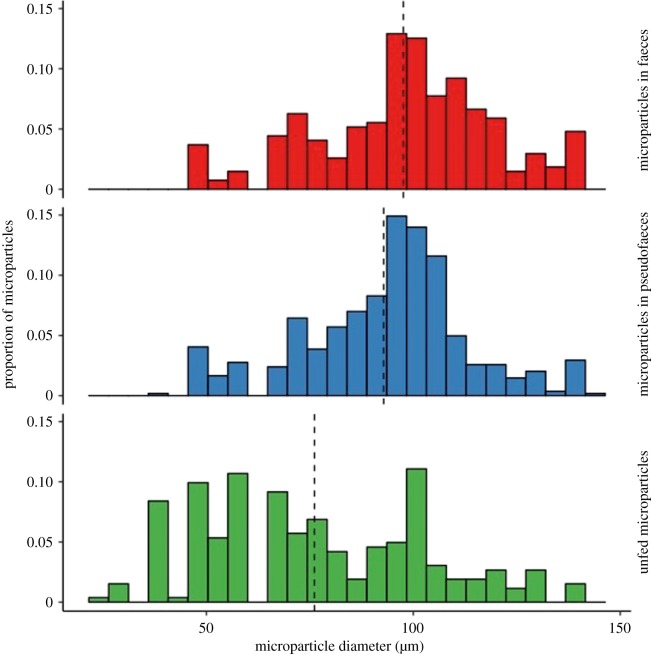
Histograms showing a greater proportion of large microparticles in faeces (red bars) and pseudofaeces (blue bars) than in unfed microparticle samples (green bars). The diameter of microparticles within faeces and pseudofaeces of eight mussels and three unfed microparticle samples was measured. Microparticle size distribution differed significantly between all sample types (least-square means, *p* < 0.001). Mean particle diameter (dotted black lines), was greatest in faeces, then pseudofaeces, and lowest in unfed microparticles (Kruskal–Wallis with *post hoc* Dunn's test, *p* < 0.001). Microparticles in each sample type: faeces *n* = 271, pseudofaeces *n* = 543, unfed *n *= 262.

A greater proportion of large microparticles was present in pseudofaeces than in unfed microparticle samples (figure 3; least-square means, *z* = 23.7, *p* < 0.001). At 92.85 ± s.e. 0.86 µm, the mean diameter of microparticles in pseudofaeces was significantly greater than the 76.15 ± 1.61 µm in samples of unfed microparticles (Kruskal–Wallis, $\chi_{2}^{2}=112$, *p* < 0.001, *post hoc* Dunn's test *p* < 0.001).

**Figure 4.**
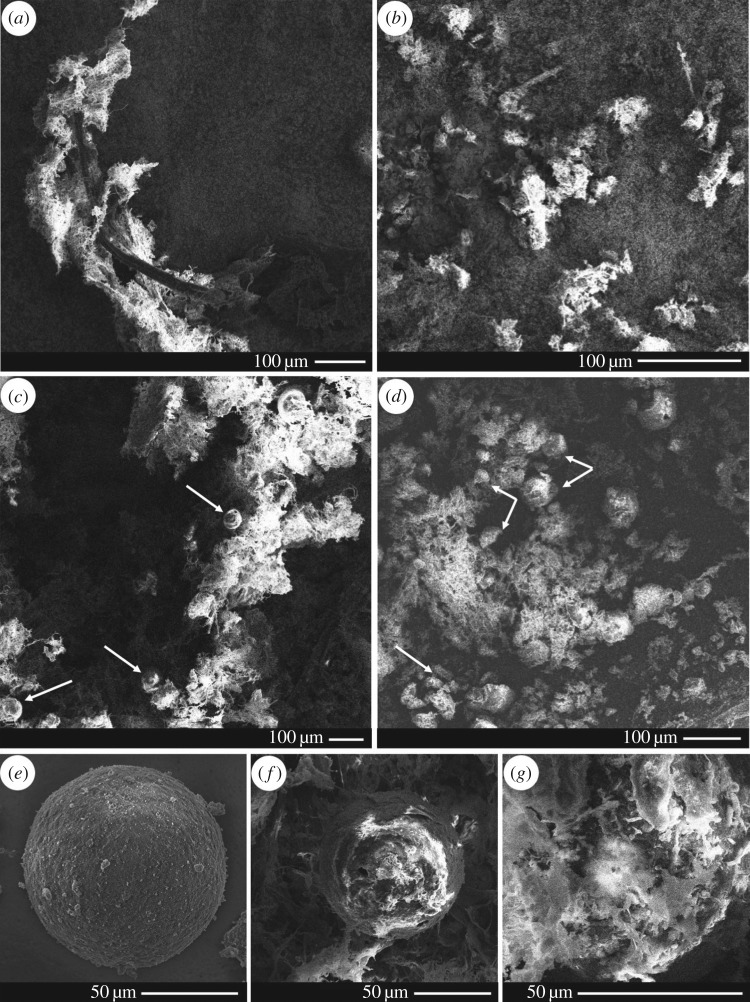
SEM images showing microparticles in mussel pseudofaeces and faeces. The following samples were collected: unfed microparticles, mussel pseudofaeces and faeces, and pseudofaeces and faeces from mussels fed microparticles. Samples were freeze-dried, carbon coated, and visualized under SEM. (*a*) Pseudofaeces and (*b*) faeces from mussels not fed microparticles. (*c*) Pseudofaeces and (*d*) faeces from mussels fed microparticles. Microparticles are indicated by white arrows. In (*c*) microparticles are morphologically similar to unfed microparticles. In (*d*) microparticles are present in a variety of degradation states; at the bottom left there is a particle which appears to be split into four pieces, and near the centre there are several particle fragments alongside multiple complete but degraded particles. (*e*) Single unfed microparticle, (*f*) single microparticle in pseudofaeces and (*g*) single microparticle in faeces.

The SEM images showed microparticles embedded within pseudofaecal threads (figure 4). These microparticles were mostly undamaged and similar in morphology to unfed microparticles.

### 3.3. Postingestive particle processing

**Figure 5.**
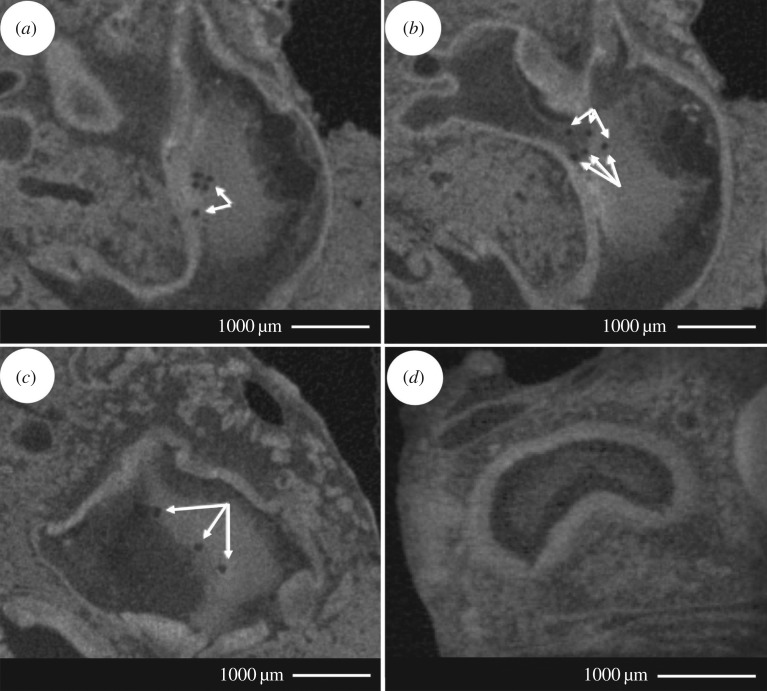
Micro-CT images of microparticles within the mussel stomach. Mussels were fed microparticles, fixed in formaldehyde, stained in phosphotungstic acid (PTA), scanned using micro-CT, then analysed using ImageJ [14]. This process was repeated for mussels not fed microparticles. The white arrows in (*a*), (*b*) and (*c*) point to microparticles of approximately 100 µm in stomach sections from mussels fed microparticles. (*d*) Stomach section from a mussel not fed microparticles.

Micro-CT scanning revealed microparticles within the stomach cavity of mussels fed microparticles (figure 5). The microparticles were of approximately 100 µm in diameter, and present across multiple sections of the mussel stomach.

Analyses of faecal samples revealed the presence of microparticles within faeces. Of the complete microparticles found in faeces samples, a greater proportion were of large diameters compared to unfed microparticle samples. Figure 3 demonstrates how the distribution of microparticle sizes differed significantly between faeces and unfed microparticles, with a much greater proportion of large diameter microparticles in faeces (least-square means, *z* = 26.5, *p* < 0.001). In addition, the mean diameter of microparticles in faeces (97.60 ± 1.33 µm) was significantly greater than in samples of unfed microparticles (76.15 ± 1.61 µm) (Kruskal–Wallis, $\chi_{2}^{2}=112$, *p* < 0.001, *post hoc* Dunn's test *p* < 0.001). When comparing faeces with pseudofaeces, the mean microparticle diameter was large in both cases, but significantly greater in faeces (Dunn's test, *p* < 0.01).

The SEM images further confirmed the presence of microparticles within faeces (figure 4). These microparticles were far more degraded in morphology than unfed microparticles or microparticles found in pseudofaeces. Completely fragmented microparticles were also seen in the faecal samples.

## 4. Discussion

### 4.1. Particle capture

The particle capture experiments demonstrated that BioBullets microparticles containing a microalgal food can be cleared from water by bivalves. This is supported by the 52.7% decline in microparticle concentration in aquaria with mussels (after accounting for background settlement), and suggests that microparticles are successfully processed during the initial stages of digestion; specifically the movement of food particles along gill filaments, into the ventral grooves and onto the labial palps (figure 1). The decline in clearance rate with falling aquaria microparticle concentration indicates that the rate of microparticle capture by mussels is proportional to the abundance of microparticles in the water. The gradual and small decay in microparticle concentration in control aquaria can be explained by the slow sinking of microparticles to the aquaria base. The particles were specifically engineered to possess slightly negative buoyancy in order to increase the likelihood of uptake by bivalve molluscs, which are found at the bottom of aquaria in commercial hatcheries.

Our investigation also indicates that BioBullets microparticles can be cleared with similar efficiency to other food sources. Firstly, natural food sources; studies on feeding greenshell mussels *Perna canaliculus* have shown that the typical decline in concentration of a standard microalgal food over 60 min, without accounting for background settlement, is 90%, very comparable to the 83.6% decline in BioBullets microparticles in our investigation (without accounting for background settlement) [16]. Secondly, microencapsulated foods; there are a very limited number of studies which have trialled the use of older lines of microencapsulated feeds including ‘MySpat’ (INVE Technologies, Dendermonde, Belgium) and ‘Frippak’ (Frippak Feeds, Basingstoke, UK) to feed bivalves. These studies assessed bivalve growth rates, and did not assess stages of digestive processing beyond particle clearance and rejection in pseudofaeces [4,17,18]. The 80% decline in MySpat concentration over 60 min caused by feeding greenshell mussel spat in these studies is again comparable to the rate of BioBullets microparticle clearance in our investigation [16]. However, while MySpat is specifically designed for juvenile mussels of around 1 mm shell length, BioBullets is more broadscale and can be used to feed larger mussels. This could be highly desirable for improving broodstock, delivering therapeutics, or for other final ‘polishing’ such as adding desirable nutrients or flavours. Additionally, an artificial diet suitable for larger individuals may be of particular benefit to other bivalve species, such as oysters, that are held in hatcheries for many years before open water grow-out.

### 4.2. Preingestive particle processing

Preingestive particle processing experiments led to two key findings. Firstly, BioBullets microparticles can be processed and rejected by the labial palps of mussels, indicated by the presence of microparticles in the SEM images. Secondly, mussels preferentially rejected larger diameter microparticles, demonstrated by the larger mean diameter of microparticles in pseudofaeces than in unfed microparticle samples. The greater proportion of large microparticles in pseudofaeces suggests the presence of a size threshold above which microparticles have a greater chance of rejection on the labial palps. Microparticles below threshold size should be ingested through the mouth (figure 1), although the efficiency of this may have been reduced in our investigations if the gills and labial palps became partially blocked by large microparticles during feeding. Both the preferential ingestion of smaller particles and the potential for larger particles to block the gills and labial palps has been shown for other artificial diets fed to *Perna canaliculus* mussels [16]. Previous studies have also shown that particle size preference of natural algal foods differs across bivalve growth stages, with mussel larvae preferring smaller particles of around 25 µm, and juveniles preferring 40 µm particles [10]. Therefore, when formulating microencapsulated foods for specific applications in the bivalve industry, it will be important to tailor particle diameter to the bivalve growth stage to avoid high rejection rates and wastage.

### 4.3. Postingestive particle processing

The postingestive particle processing experiments demonstrated that BioBullets microparticles were ingested by mussels, and that microparticles could be broken down by the mussel gut. Ingestion was demonstrated by the presence of microparticles in micro-CT scans of the mussel stomach, and successful microparticle breakdown was indicated by the presence of degraded and fragmented microparticles in faeces. The finding that most microparticles present in faeces were of large diameters compared to unfed microparticle samples again suggests the presence of a threshold particle size, above which digestion in the stomach becomes problematic. Any large microparticles that are accepted through the labial palps are probably being channelled from the ciliated sorting areas to deep rejection grooves in the stomach, as also occurs with large natural food particles [12] (figure 1). They then pass into the intestines and are excreted, leading to a high proportion of large microparticles in faeces. In comparison, smaller microparticles are successfully broken down by digestive enzymes released due to the rotating action of the crystalline style, kept in suspension, and swept towards digestive gland ducts for absorption.

## 5. Conclusion

Our study has demonstrated that a new form of microencapsulated diet known as BioBullets can successfully be ingested by a commercially farmed bivalve, the blue mussel. With further work, this could open up numerous opportunities for the application of novel microencapsulation technologies in the bivalve shellfish industry. There is a need for future investigations to demonstrate that the contents of ingested BioBullets can be assimilated by bivalves and used to fuel anabolic processes, by comparing the growth responses of bivalves fed BioBullets to those fed standard diets. This study highlighted the importance of developing and feeding microencapsulated diets of an optimal size to bivalves, to avoid high levels of rejection in pseudofaeces or faeces. The chemical engineering approach can allow us to tailor the size of particles to the feeding preferences of specific bivalve species or growth stages. There is also a need to understand the optimal formulation of nutrients to encapsulate within microencapsulated diets. This would enable us to enhance the growth and conditioning of specific bivalve species or growth stages, or improve bivalve growth in geographies where key nutrients are lacking.

There is therefore considerable opportunity in developing the BioBullets system as a method to deliver highly nutritious microencapsulated diets to bivalve shellfish. Microencapsulated diets have potential to significantly reduce bivalve production costs, increase production output, and contribute to the continued growth of bivalve aquaculture.

## Supplementary Material

Particle Clearance Spreadsheet

## Supplementary Material

Particle Size Selectivity Spreadsheet

## Supplementary Material

Particle Clearance R Script

## Supplementary Material

Particle Size Selectivity R Script

## Data accessibility

The datasets supporting this article have been uploaded as part of the electronic supplementary material.
